# Perceived impact of community kitchens on the food security of Syrian refugees and kitchen workers in Lebanon: Qualitative evidence in a displacement context

**DOI:** 10.1371/journal.pone.0210814

**Published:** 2019-01-25

**Authors:** Nadiya Ibrahim, Gladys Honein-AbouHaidar, Lamis Jomaa

**Affiliations:** 1 Rural and Community Development Program, Faculty of Agricultural and Food Sciences, American University of Beirut, Beirut, Lebanon; 2 Rafic Hariri School of Nursing, American University of Beirut, Beirut, Lebanon; 3 Refugee Health Program, Global Health Institute, American University of Beirut, Beirut, Lebanon; 4 Department of Nutrition and Food Sciences, Faculty of Agricultural and Food Sciences, American University of Beirut, Beirut, Lebanon; Northumbria University, UNITED KINGDOM

## Abstract

Community kitchens (CKs) have been recommended as public health strategies with social and nutritional health benefits for low-income participants and their families in different settings. The benefit of CKs in improving the food security status of participants in the context of conflict and displacement is less conclusive. This study aimed to qualitatively explore the impact of CKs on the food security status of community kitchen workers (CWs) and Syrian refugee (SR) families in Lebanon. An exploratory qualitative descriptive approach was adopted. Focus group discussions were conducted with 15 CWs and 49 SRs, and transcripts were analyzed thematically. Emerging themes included: *motivation to join the CKs* (CWs only*)*, *perception towards CKs*, *impact of these CKs*, and their *sustainability* (both groups). Motivating factors for CWs included financial, internal and societal drivers, and the favorable type of work in kitchens. The perception towards CKs was overall positive among CWs and SR beneficiaries. Both groups reported the positive impact of CKs on their food security and financial status, which in turn affected positively their psychological health. At the social level, CWs indicated that the kitchen’s friendly atmosphere increased social cohesion and companionship between Syrians and Lebanese within the kitchen. In addition, CWs reported increased sense of empathy towards SRs benefiting from the CK services. According to study participants, the positive impact of the CKs was almost completely reversed when their operation and services were interrupted for two months. Both CWs and SRs identified facilitators and barriers that can affect the sustainability of the kitchens, including financial and entrepreneurial skills. In conclusion, findings from this study highlight that CKs can be promising programs to improve the food security and livelihoods of participants, while also increasing social cohesion and integration of refugees within host communities in protracted crisis contexts.

## Introduction

Community kitchens (CKs), by definition, are small groups of people in community-based cooking programs who meet regularly to prepare one or more meals together [1]. These kitchens are commonly “characterized by the pooling of resources and labor to produce large quantities of food”, [1] and food is usually cooked for at least three to six meals a month [2].

While the descriptions and structure of CKs vary considerably in the literature, they all aim at empowering those who are vulnerable to hunger and food insecurity [2]. CKs originally developed in the 1980’s as part of “grass-roots” movements that took place mostly in urban settings around the world by women who met to cook food in bulk in order to save money and stretch their budgets [1,3,4]. A variety of CKs, or what is also known as collective kitchens, emerged since then. Health professionals, community workers, and public health authorities adopted these kitchens to promote nutrition education and alleviate food insecurity among low-income households and communities through enhancing their cooking, coping and budgeting skills, and increasing the diversity of food in their diets [1,5]. These kitchens have also been commended for empowering participants and increasing their self-reliance and dignity through making them less dependent on charitable organizations and encouraging participants to develop their own social support groups and networks within the CKs, hence reducing their feelings of social isolation [2].

Although there is consensus of the positive value of CKs, evidence on the impact of these kitchens in alleviating food insecurity is still needed particularly in low-and-middle-income countries (LMICs) and protracted crises settings. A systematic review by Iacovou and colleagues (2012) showed that CKs serving low-income families in high income countries, such as Canada, Australia, and Scotland, may play an important role in enhancing the social interactions as well as improving the cooking skills and nutritional intake of individual participants and their families [5]. Yet, reviewers argued that evidence on the impact of these kitchens in alleviating food insecurity is still needed prior to recommending them confidently as effective public health strategies [5]. Fewer studies have explored the impact of CKs on the food security and household economics of low-income families in LMICs. Instead, these studies that were conducted mostly in Latin American countries were focused on the role of CKs, as grass-roots efforts and self-help programs, in women empowerment and activism [4,6,7]. To our knowledge, there is no literature on the role and impact of CKs in alleviating food insecurity within conflict-affected and displacement settings.

Today, the Syrian refugee (SR) crisis represents one of the main humanitarian challenges and protracted crises facing the world with more than 5.5 million individuals seeking refuge in neighboring countries, including Turkey, Lebanon, and Jordan [8]. With approximately one million SRs within its borders, Lebanon has the second-largest population of refugees in the region and the highest per capita refugee population worldwide [9]. Displaced Syrian families in Lebanon are facing increasing challenges due to insufficient humanitarian funding, limited employment and livelihood opportunities, as well as the depletion of resources and assets. These conditions have been associated with high rates of food insecurity reaching 91% of the SR families and with a deterioration in the diet diversity of households [10]. To address these challenges and alleviate food insecurity, humanitarian organizations mobilized several interventions and programs in Lebanon in response to the high refugee influx. Small-scale CK that existed in few regions across the country, prior to the Syrian crisis, represented one of these projects. These kitchens aimed at providing food assistance to vulnerable SRs while also securing an employment opportunity to low-income Lebanese and SR women from the local communities. Despite anecdotal evidence in support of this food aid modality [11], the impact of these kitchens on the food security status of participants and beneficiaries in this context of conflict and displacement has not been adequately explored.

This study aimed to explore the impact of CKs in reducing hunger and food insecurity among refugees and host communities in a protracted crisis setting. More specifically, the study’s objectives were to qualitatively: 1) explore the perceptions and experiences of CK workers (CWs) and SR beneficiaries towards CKs in Lebanon, and 2) examine the impact of CKs on the food security status of study participants and their families

## Methodology

### Study design

An exploratory qualitative descriptive approach was adopted in the present study. Given the paucity of literature describing the role of CKs and their impact on the food security status of vulnerable groups in the context of conflict and displacement, we opted for an exploratory descriptive approach. This method provides a thick contextual description of the phenomenon, as experienced by study participants, and it is based on the general principles of naturalistic inquiry that has a broad range of choices for theoretical or philosophical orientations, sampling techniques and data collection strategies [12]. A conceptual framework outlined by Freedman et al. (2013) guided the data collection and analysis in the present study [13]. The study was conducted in accordance with the Declaration of Helsinki, and the ethical approval was secured from the Institutional Review Board at the American University of Beirut prior to recruitment and data collection.

### Population, eligibility criteria, and recruitment approach

Two groups constituted the target population: community kitchen workers (CWs) involved in the preparation of food and SR families receiving food assistance in the form of ‘food pots’ (cooked food). The target population was recruited from four CKs located in four different areas within two regions in Lebanon known to have the largest population of refugees in the country: the North of Lebanon (26% of refugees) and the Bekaa (36%) [9]. Eligibility criteria for CWs included: adult women (18–65 years), either Lebanese or Syrian nationality, and being involved in the local CK for at least six months. As for the SR group, criteria included being a Syrian woman of child bearing age, having at least one child, living in an Informal Tented Settlement (ITS), and received/or currently receiving food assistance in the form of hot pots from a local CK for at least six months. Based on previous studies [1,14], the six months cut-off point considered in the present study was set to be the minimum period to observe changes in household finances and food security status after receiving food assistance.

A purposeful and geographical variation recruitment approach was used. To recruit CWs, the research team first contacted the administrations of CKs to get their preliminary approval prior to arranging for a site visit to meet with the CWs. As for SR women, the research team contacted the humanitarian agency involved in delivering ‘food pots’ to the beneficiaries in order to identify the location of their ITSs. The CK administration and humanitarian agency did not have any direct or indirect role in the recruitment of study participants or access to any of the collected data. Direct recruitment approach was used on site to invite women from both the CWs and SR groups to participate in the Focus Group Discussions (FGDs).

### Conceptual framework

A conceptual framework previously outlined by Freedman et al. (2013) was adopted in the present study [13]. This multicomponent model was originally developed to assess the access of individuals to nutritious food in the United States, and it was based on five main constructs: economic aspect, service delivery, spatial-temporal features, social aspects, and personal aspects [13]. We used these constructs when developing the two topic guides for our focus group discussions with the study participants: one for CWs (see S1 Table) and second for the SR group (S2 Table).

### Data collection

Data collection for the study took place between July and October 2017. A total of eight focus groups were conducted during that time period after which we reached data saturation: four with CWs and four with SRs. FGDs with CWs were held between July and August 2017. During that period, the CKs were not operating due to fund interruptions, yet they were expected to resume work beginning September 2017. FGDs with the SR group took place between September and October 2017. During this period, CK services had resumed again, but the implementing organization selected new ITSs and different families to participate in the new phase of the CK project. Therefore, in order to meet the six months inclusion criteria of our study, we chose to collect data from ITSs that were involved in the CK project prior to the two-month-interruption of the kitchens in the summer. Thus, the recruited SR families were not receiving services from the CKs at the time of data collection.

The researcher (NI) received training on the ethical and responsible conduct of research and FGD moderation prior to data collection. NI moderated the FGDs and the P.I. (LJ), co-P.I. (GHA) and Research Assistant (MDH) were present as observers and took written and mental notes. All focus groups were audio-recorded after securing the consent of study participants. Verbal consent of the participants was obtained in case the participant was illiterate; a witness assigned by the participant explained the content of the oral consent form as well as the objectives and activities of the projects in a culturally sensitive, easy and comprehensible language and manner.

After obtaining participants’ consent, the moderator proceeded to circulate a short survey that included questions related to the demographic and socio-economic characteristics of the study participants, such as age, marital status, educational attainment, employment status, family monthly income and expenditures, and form of assistance received in the past three months, if any. In addition, crowding index was calculated, as a proxy measure for the socioeconomic status of households, through dividing the total number of household members by the total number of chambers in a household (excluding kitchens, bathrooms and balconies). This index was previously used to assess the socioeconomic status of refugees in Lebanon and similar contexts showing reliable results [15–17].

### Data analysis

Data collected from the short questionnaire was analyzed using SPSS 19 software. Descriptive analyses included means and standard deviations for continuous variable sand frequencies and proportions for categorical variables. As for the analysis of the FGDs, thematic deductive analytical approach was adopted. All FGDs were first transcribed verbatim in Arabic (language used to conduct the focus groups). The transcripts reflected the factual account of the discussion including the pauses, laughter, and emphases. Each participant was given an ID on the focus group (e.g. FG1P1). For the SR group and due to voice interference with children being present at the time of the focus groups, we couldn’t identify the different participants in the recorded discussions; hence, the identifiers did not include the participant’s number (e.g. FG1). The second step was conducting the analysis using a thematic approach framed by Freedman’s conceptual framework. The thematic analysis consisted of 6 phases: phase 1, two researchers were immersed in the data by reading and re-reading each transcript in order to familiarize themselves with the information provided; phase 2, the data coding started and was based on the concepts of Freedman’s conceptual framework (economic, service delivery, spatial–temporal features, social and personal aspects) (Open coding); phase 3, the list of codes were refined through discussions among the two researchers and with the P.I. in order to identify the links between them; In phase 4, themes that match the framework and those that expand or merge with existing concepts were identified along with the associated sub-themes (axial coding); in phase 5, the final themes and subthemes were refined through discussions; finally in phase 6, a complete narrative of the findings was provided [18]. The research team supported these findings with relevant quotes for each theme and sub-theme. Deviant cases were included in the final narrative and noted as “one”, “a couple”, or “a few participants indicated”, hence giving voices to singular opinions. The observation notes and interviewer’s mental notes from the focus groups were maintained to describe the interactions that surrounded the focus group discussions.

#### Increasing rigor

The research team ensured that credibility and reflexivity were observed. In terms of credibility, the research team relied on the transcribed, audio-recorded interviews as the main data repository supported by mental notes and the observers’ notes. In addition, each theme and sub-theme was supported with quotes. Every attempt was made to maintain a level of reflexivity at each stage of the research. At the pre and during data collection phases, the research team made sure to avoid any undue influence on the participants. Prior to the start of the data-collection, both the moderator and the observer had undergone bracketing, outlining their positions and personal assumptions before the first focus group [19]. During data collection, both mental and written notes were taken regarding participants’ transparencies, preliminary emerging themes and whether it was felt that participants were influenced by the moderator. During data analysis, frequent team meetings were conducted, where emerging themes were compared and contrasted, putting primacy to the collected data. The personal assumptions that the team had during bracketing were revisited at that time, and the team reflected on whether those personal views and assumptions may have influenced the interpretation of the data and acknowledged their influence on the findings. In addition, the research team followed the COnsolidated criteria for REporting Qualitative research (COREQ) checklist for reporting this study (see S3 Table) [20].

## Results

### Demographic and socio-economic characteristics of study participants

A total of 15 participants were from the CWs group and 49 were from the SR community. All participants were women and the majority were married (n = 53) with an average age of 36.9 ± 10.7 years. A total of 40 participants completed at least primary level schooling, yet only few completed secondary education or higher (n = 14). Significant socio-economic differences were noted between both groups (CWs and SR women) in the present study. CWs had higher monthly income (93.3% vs 19.6% earning ≥ $ 200, p<0.001) and higher total monthly expenditures as compared to the SR group ($714.7 ± 400 vs. $318.6 ± 171, p = 0.002). On the other hand, SRs had on average higher crowding index (4.2 ± 2 vs. 2.3 ± 2, p = 0.002), reflecting lower socio-economic status, and higher dependence on the World Food Programme (WFP) e-card assistance as compared to the CWs (66.7% vs 13.3%, p<0.001, respectively). In addition, 39 out of 49 SRs in the study sample reported severe food insecurity, whereas only 6 out of 15 CWs reported a severe level of food insecurity (p<0.001).

### Emerging themes

**Fig 1:**
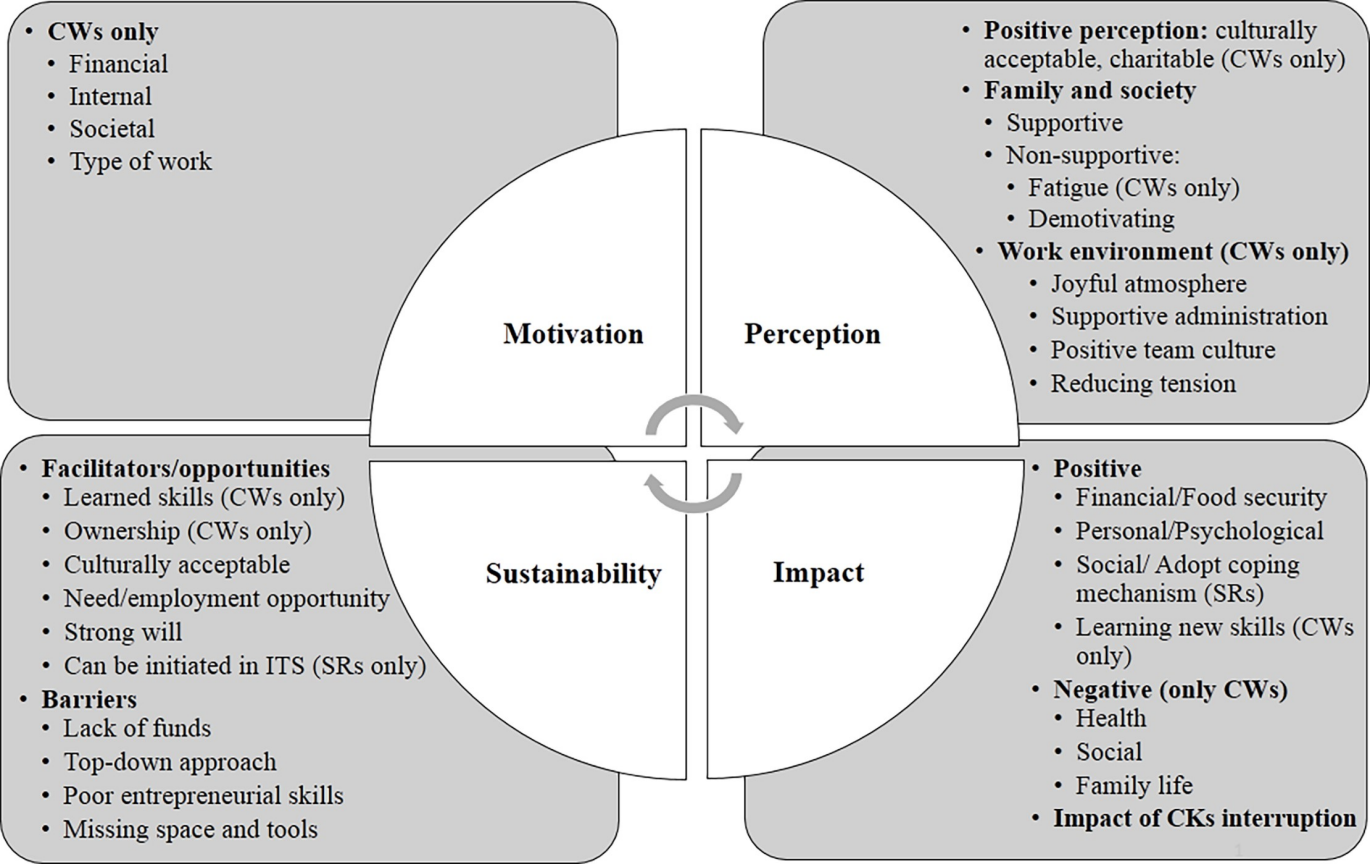
Summary of main themes and subthemes from FGDs with CWs and SRs.

The thematic analysis from the focus groups revealed four main themes that describe the experience with CKs: a) motivation to work in CKs (among CWs group only), and three themes that were in common between CWs and SRs, namely b) perception towards CKs, c) impact (impact of CKs and the impact of the CK interruption, and the d) sustainability of CKs. Within each theme, we identified several sub-themes (see **Fig 1** for a graphical representation of the emerging themes).

**a) Motivation to work in CKs**

CWs pointed to financial, internal, and societal drivers for participating in CKs. Some CWs also reported being motivated by the type of work. With respect to the *financial drivers*, being the head of the household and the *“high cost of living” (FG3P1*, *FG3P3)* were the two major reasons that led CWs to work in these kitchens. CWs needed the income to attend to their family needs. In addition, financial independence and avoiding charity motivated them to secure a stable monthly income. *“I don’t ask anyone for assistance and I don't wait until someone knocks at my door to help us*. *I always try to avoid dependency even if it was at the expense of my health and wellbeing*, *or even my time” (FG1P1)*.

One of the main *internal drivers* that motivated many CWs to participate in CKs was the urge for *altruism*: *“I was not working in the CK only for the sake of getting paid*. *My goal was to help the Syrian refugees*. *We are ready to help them to the best of our abilities”* (FG4P3). For other CWs, *self-development* was another internal driver for working in CK. Their participation in these kitchens allowed them to gain experience and achieve self-realization: “*I feel more confident when I work*. *I can feel my self-worth when I receive an income at the end of the month and that’s probably not only due to financial reasons*” (FG1P1). Some CWs also reported that working in the kitchens enabled them to make the best use of their free time and to organize their life better.

With respect to the *societal drivers*, CWs pointed that their participation in CKs improved their social life and reduced their sense of loneliness *“I thought I would feel happy spending some time with companions here because at home I’m lonely all the time” (FG4P4)*. Also, some expressed that their involvement in the CKs made them feel socially valuable.

The *type of work* was also a motivator for some CWs due to factors such as convenience, the enjoyable nature of cooking and the loyalty of some kitchen workers to the kitchen. One CW acknowledged that the team is very passionate about cooking: “*We love cooking and our food smells amazing*! *We tell everyone who comments on the smell of our cooking that we are not just working to get paid at the end of the month*, *we cook with passion*!” (FG1P1). In terms of convenience, most CWs reported living near the kitchens, which also motivated their choice of work. In addition, few women stated that cooking is the most convenient type of work for housewives.

**b) Perception towards CKs**

One of the themes that emerged from the focus groups with the CWs and SRs was an overall positive perception towards CKs. CWs considered working in kitchens to be a culturally acceptable type of work: “*cooking is a female job*” (FG4P2). It was also perceived as charitable work helping many refugees, thus it was “an honorable job”: *“People used to say that we should be proud of ourselves because we were doing a charitable work” (FG3P1)*. In addition, most CWs reported that their families and local communities supported them showing more respect when they started working in the CKs “*People showed us more respect because we started supporting more families in addition to the families we used to support before the CK project*” (FG4P4). Such a supportive and positive attitude towards CKs motivated other women within their local communities to consider joining the cooking team.

Although the majority of SRs were familiar with the concept of CK, which emerged mainly during the war period in Syria over the last few years, a few said that it was a new experience for them. Overall, SRs appreciated the CK concept and services and expressed their interest in implementing similar kitchens within their local communities once they return back to Syria.

CKs were negatively perceived by only few CWs and SRs. Among CWs, some pointed to fatigue *“Our work is physically demanding indeed and we feel extremely tired especially during the month of Ramadan"(FG3P1)*. Few SR women in one of the visited ITSs indicated that the process of choosing families to benefit from CK was neither transparent nor fair, causing confusion and dissatisfaction among the refugee community: “*My neighbor is a school teacher and her husband is a full-time employee and she was registered for the project*!*”* (FG3).

CWs agreed that the atmosphere in the kitchens was overall positive and joyful: “*We used to have a lot of fun here*. *The atmosphere was always joyful*” (FG4P3). Other positive aspects of the work environment included supportive administration and positive team culture, which strengthened social cohesion between the workers from different religious and cultural backgrounds (for example between the SR and Lebanese women working in the same kitchens). CK workers emphasized that they spent most of their time together in the kitchen, which strengthened the bond between them and reduced tension and conflicts among team members. A sense of trust and confidentiality between the CWs developed, which provided them with needed psychological support. Finally, some women reported that the overall positive environment created a sense of belonging to the kitchen, where they felt like they are all part of one family.

**c) Impact of CKs**

This theme was further divided into two subthemes: 1) impact of being involved in the CK services and the impact of CK interruption. With respect to the impact of CK services, both groups considered CKs to have a positive impact on the food security, financial, personal/ ] psychological, and societal aspects of their lives. In addition, CWs reported learning new skills due to their involvement in these kitchens.

CKs provided both groups with food pots on a regular basis, which helped meet their needs for food and cut down on their food expenditures. Almost all the SR beneficiaries included in the present study reported that CKs fulfilled a dire need for food for all family members, especially for children. CKs allowed them to consume a wider variety of food items and in larger quantities, and that this improved their nutritional and health status. “*It was an opportunity for us to consume foods that we couldn’t afford on a weekly basis such as beef and chicken*” (FG3). Another Syrian woman reported: “*We used to be weak and our children were slim*. *When we started receiving food*, *our health became much better*” (FG4).

In terms of the *financial implications* of the CK, the food pots had a cost opportunity impact. They allowed the families of both groups of women to cut their spending on food in order to buy other necessities such as medicine and fuel: *“The food pots that I took to my home helped me cut the spending on bread and food" (FG4P2)*. “*We used the money that we saved to cover our house rental fees and electricity bills*” (FG3). Most CWs indicated that the steady monthly income they received in return for their work with the CK helped them achieve financial independence and preserved their dignity by not having to rely on charity: “*The kitchen supported me a lot*. *I used to have headaches before and my brother used to take me to the doctor and pay the fees*. *Now it's different*. *I don’t ask anyone for help*, *even if it was my son*. *I am still able to work and be productive and I don’t want to be dependent on anyone*” (FG1P2). In addition, most SRs reported that the CK services saved them from falling into debt.

CWs and SR participants reported *personal and psychological benefits* from their involvement in the CK. The kitchens provided CW and SRs with a “*peace of mind*” by not having to worry constantly about their food security and how to provide food for their families. SR noted that by receiving food pots, they were able to dedicate more time for other activities, including house chores, sleeping, or socializing. CWs noted that the financial independence gained from participating in the CK empowered them psychologically “*The kitchen did really give us strength and motivation*” (FG3P2).

The *social capital impact* that the CK gave to CW was unequivocally stated. The kitchens gave them the opportunity to socialize with fellow workers inside and outside the kitchens *“Now we have stronger relations with each other also outside the kitchen”* (FG3P3). A few CW only indicated that their work in the kitchens limited their time to socialize outside the kitchen. As for the social impact of the CK on SRs, study participants reported that having the same food pots served to all beneficiaries created a sense of unity, agency and equity within their community: “*We were satisfied and content because we were all eating the same type of food*. *Previously*, *the smell of cooking from our neighbor’s house used to make us crave for food that we couldn’t afford buying*” (FG4).

Another impact of the CKs noted among CWs was *learning new skills* for food preparation and financial management. CWs reported acquiring a variety of new skills such cooking food in bulk amounts, preparing a diversity of new dishes, and learning and practising basic food hygiene and food safety principles. Learning important financial management skills were also noted among CWs: “*My Colleague told me things about how she managed the money she had on hand*. *I listened to her because I learned from her*. *When I listen to others*, *I become more aware of how to manage my finances*” (FG1P1).

Despite the overall benefits of working in CKs, few CWs reported that their participation in these kitchens had also some negative impact on their health, family time and social life and family time. Some reported that working in CKs caused tiredness leading to the emergence of new physical conditions and the worsening of already existing physical conditions. In addition, some CWs who have young children reported that working at the CKs limited the time they could spend with their children, which was a negative impact of CKs. Few CWs reported that their work in these kitchens limited their time for socialization, while others disagreed on whether this is considered a drawback of their work, as they viewed it as a positive matter: “*I don’t consider this as something negative*. *Sometimes when I used to see people around me only interested in gossiping*, *I felt very thankful that I had my own interests and that my time was organized*. *The most important thing is that your family is doing well*” (FG1P1).

The second sub-theme under the impact of CKs that emerged during the FGDs with women from both CW and SR groups was the impact of having the CK services being interrupted during the summer months prior to the data collection, and its negative implications on their household finances, food and nutrition security status, and their overall psychological health.

In terms of the *financial and food security implications*, both groups stated an increase in their financial burden when CK services were interrupted, which resulted in spending all saved money among CWs and the failure of the SR beneficiaries in fulfilling their family’s basic needs. In addition, the majority of SRs in the study reported exacerbated debts and loss of cost opportunity to purchase something else during that period: “*We had to spend all the money that we saved before because we are jobless*” (FG4P4), “*I feel weak because I don’t have money and I am worried about providing for my children*” (FG1). During the interruption of the CK, the amount of food available for individuals to consume decreased among families in both groups, particularly among SRs. They reported that food quality and quantity deteriorated among household members, which negatively affected their nutritional and health status, mainly children: “*We used to consume meat 2 times per week*. *Now we can afford it only twice per month*” (FG4); *“Recently*, *my children started to look pale and I couldn’t figure out the reason*. *So I took my child to a health center and his blood test showed that he’s anemic*” (FG1).

In terms of personal and psychological health, overall, the discontinuation of kitchens had a negative psychological impact on all study participants and this impact was more pronounced among the SR group. Women from both groups reported feeling helpless and depressed as a result of the interruption of kitchen services: “*For me*, *there's no life anymore*. *I don’t know if it's the same for others*. *Although I don’t have any family responsibilities*, *I'm really in need*. *I cry many times because I feel sorry for myself and I try to hide it from my parents*. *I’m the one who’s suffering the most*” (FG4P3). “*We became psychologically and financially distressed*” (FG2). CWs and SRs reported that the halting of the CK services affected their social lives and daily routines. For SRs, the disruption of the kitchen services created tension within their families: “*I have four children*, *together with my husband we are six*. *We have to pay 200$ house rental fees and almost 65$ electricity charges*. *I am left with only 260$ which I have to use wisely and this creates tension inside my home*” (FG3). Women had to deal mostly with this difficult situation and they reported adopting different coping mechanisms such as saving food for children and depleting limited assets. Similarly, CWs had to think of alternatives, such as searching for a different job or urging their respective kitchen’s administration not to stop. It was also noted from the FGDs with SRs that the halting of the CK services adversely exacerbated social tension between refugees with the local host community and caused confusion as to what services are offered to each of the groups: “W*e [SR] ask them [non-governmental agencies] to give us assistance unit but they tell us you are Syrians and this assistance is only for the Lebanese*. *They give them meat*!” (FG3).

**d) Sustainability of CKs: Facilitators and barriers**

Both CWs and SRs showed interest in the sustainability of the CK model. SRs, in particular, showed interest in initiating SR-led kitchens inside their ITSs, where the whole community would be able to participate in activities aimed at food provision.

Women from both CWs and SR groups also identified facilitators/opportunities, barriers, and threats for the sustainability of the CKs in their current set-up. In terms of the *facilitators and opportunities* for the sustainability of the CKs, both groups reported that having the basic cooking skills is a major facilitator for their involvement in CK. Among CWs, the sense of belonging and “ownership” of the kitchens were perceived as important factors for moving forward: *“We were among the founding members when the cooperative was officially registered” (FG1P1)*. Participants noted that access to funding from non-governmental organizations (NGOs), can be an opportunity for them to ensure the continuity of their local CKs. The cultural acceptability of this type of work for women was also identified by participants as an opportunity that allows them to expand on the current CK model. Opportunities for SR women to start CK initiatives within their communities included catering for big events that take place within the ITSs, such as weddings and funerals. As for CWs, the suggested business options included farmer’s markets where they can prepare *Mouneh* (local and traditional food items), frozen vegetables, desserts and pastries, and frozen *Mezze* items (small-sized Mediterranean appetizers), in addition to catering for events.

With respect to the *barriers and threats* that may limit the continuity of the CKs, both groups (CWs and SRs) mentioned the lack of personal funds as a major barrier for moving forward if the CK are no longer supported by funding from humanitarian organizations. A CW reported *“You know we all have limited resources*, *if someone supports us with funds*, *we would start working and selling our produce” (FG1P3); whereas a SR stated* “*We like the idea of initiating a CK however funds are missing and we don’t have the guts to propose this idea to donors*” (FG4). Other barriers reported amongst CW were their limited entrepreneurial and leadership skills or training to be able to move forward “*Another obstacle we used to struggle with was the problem of marketing our products” (FG1P1);* “*We don't have a say in this because the kitchen administration is the final decision maker*” (FG3P1). In addition, CWs mentioned that because CKs are NGO-driven, the threat to their sustainability was bound to happen at any time. The financial support of NGOs was crucial for space, tools, ingredients, and distribution. Without this support, they cannot have a sustainability plan.

For additional quotes supporting themes and sub-themes depicted from focus groups with CWs and SRs respectively, please refer to Tables 1 and 2.

**Table 1:** Complete list of themes, subthemes and supportive quotes for community workers (CWs).

Main theme	Subthemes	Supportive quotes
**a. Motivation to work in CKs**	**1. Financial drivers:** - Need (family/household, husband not working, head of the household, expensive cost of living) - Financial independence (stability, avoid charity (family and personal)	- *“Nowadays*, *it is necessary that two family members share household expenses*, *since the income of one member is not enough to cover family needs*, *especially if there are children*. *You know*, *securing a decent living for your family and educating your children is not an easy job” (FG3P3)*. *- “I used to work in agriculture*, *and when the kitchen was established*, *I decided to participate as a community worker because here I can get a stable monthly income” (FG1P4)*.
	**2. Internal drivers:** - Altruism (love to do volunteer worker, helping others (Syrians)) - Self-development (benefiting on the personal level, self-realization and gaining experience) - Enjoy working (making use of wasted time, organizing their life, satisfaction (mutual benefit, positive feedback))	- *“My goal behind participating in the community kitchen was not only to achieve an income*. *I was happy working as a volunteer for our cooperative*, *where we used to serve our community before we got involved in the community kitchens project*. *We love to do charity work” (GF4P1)*. - *“Through participating in the community kitchen*, *I can feel my self-worth because working allows me to take a share of family expenses” (FG2P1)*. *- “If we don't work*, *we would stay at home*, *do household chores and then we would have plenty of free time*. *Through working in the kitchen*, *we can make use of wasted time” (FG1P3)*.
	**3. Societal drivers:** - Benefiting the society - Enjoy going out/companionship	- *“I like to fulfill my role in the society” (FG1P2)*. - *“I personally love going out” (FG1P1)*.
	**4. Type of work:** - Enjoy cooking - Convenience - Loyalty to that specific kitchen	- *“Cooking is my hobby*. *It is all about loving what you do” (FG1P1)*. - *“This is the most convenient type of work for housewives” (FG4P2)*. - *“My colleague and I are working here since many years*. *In fact*, *we started working in this organization 20 years ago" (FG3P1)*.
**b. Perception towards CKs**	**1. Positive perception:** (type of work, work environment, more women asking to join)	- *“Many people asked us to inform them whenever we needed more employees” (FG2P1)*.
	**2. Family and social perception for community kitchens** - Supportive: ○ Family/society is supportive (proud of the CWs) - Non-supportive family and society (few CWs) ○ Family can be non-supportive especially when the CWs feel tired ○ Society is sometimes demotivating	- *“My parents were so happy when they found me this job” (FG4P3)*. - *“My children prefer that I stay at home and don’t work” (FG3P2)*. - *“There were people who demotivated me by asking what thing I get from working while having to leave my house and children*. *They saw me looking tired and asked why they weren’t seeing me more often*. *I said I was tired because I was working*, *then they replied*: *“why do you need to work*! *Stay at your home it’s better for you to settle down…” (FG1P1)*.
	**3. Work environment** - Overall joyful atmosphere - Supportive administration - Positive team culture ○ Cultural exchange ○ Social cohesion between Lebanese and Syrians, religious groups ○ Learning positive attitudes ○ Psychological support ○ Like a family, friendly environment, love the time spent together, no conflicts ○ Trust and confidentiality ○ Sense of belonging • One center indicated that in the beginning there was some tension between Syrians and Lebanese but was resolved overtime	- *“We didn’t work all the time*. *For example we sat to talk and laugh together*. *We also prepared lunch and had it together with the kitchen administration” (FG3P3)*. - *“Honestly*, *the kitchen administration didn’t act bossy*. *On the contrary*, *we worked together as one team*. *The kitchen responsible helped us in our work*, *especially in the first phase where everything was new to us” (FG2P1)*. - *“There are many things they have in their culture that our culture lacks*. *So*, *there was an exchange of experiences” (FG4P1)*. - *“We have zero intolerance among us” (FG3P3)*. - *“She [pointing to one of her colleagues] respects my religion and I respect her beliefs*. *We never had any religious or political talks inside the kitchen” (FG3)*. - *“We learned positive attitudes about life” (FG1P2)*. - *“The work environment in the kitchen was giving us a bit of security and psychological relief” (FG3P3)*. - *“We are friends more than colleagues” (FG2P1)*. - *“We shared our personal stories with each other; what was going on in our lives and what things we were doing” (FG2P1)*. - *“For me*, *I feel I owe the orphanage a lot*. *It has supported me on the personal level*. *It has given me the strength and also helped me raise my children*. *I love this place a lot*. *I love my workplace” (FG3P2)*. - *“In the beginning*, *there was tension between us and you could feel it*. *Then with time we were able to adapt*. *I can’t say it was easy for us to accept each other*. *I am honest and I say things in a direct way” (FG4P1)*.
**c. Impact of CKs**	**Impact of being involved in the CK services** **• Positive** **1. Financial support/food security:** - Income saving on family food expenses, financial independence, dignity (not having to rely on charity)	- *“The kitchen was supportive*. *We were able to make ends meet as the kitchen somehow helped us save on expenses” (FG2P3)*.
	**2. Personal/psychological:** - Satisfaction (life saver, financial independence) - Empowerment (autonomy in decision making)	- *“Having a job is very necessary*. *I felt proud of myself because I no longer had to ask someone for money when I wanted to go out or buy meat*, *vegetables or anything else for my house” (FG4P4)*. - *“Everything I do now is a result of my own decision and nobody has a say in it” (FG4P4)*.
	**3. Social:** - Improved social life (Social relations: with colleagues outside the kitchen, social relations with other beneficiaries) - Motivating others to work within CKs - Altruistic work, empathy towards the CK beneficiaries (feeling thankful and blessed when looking at their situation)	- *“My colleague and I walked to our homes together because we both live in the same area*. *On our way*, *we met people who were receiving our food*. *They used to thank us and give us feedback on the food” (FG3P1)*. - *“As soon as the project was launched and people began to see our work*, *they started to come and ask to join us” (FG1P3)*. - *”You might pass through tough times once in your life*, *maybe twice*, *but there are people who are always having hardships” (FG2P1)*.
	**4. Learning new skills:** - Kitchen skills (cooking bulky amounts, food hygiene and food safety principles, cooking new types of dishes, washing big amounts of pots and utensils) - Financial management (valuing money, learning how to save money for the times of need)	- *“Also with respect to cooking*, *we learned from each other how to cook new dishes from both the Lebanese and Syrian cuisines” (FG4P4);“There are simple food handling tips that we thought we were aware of or that we knew but we didn't apply*. *For example*, *they told us that cooked food should not be left unrefrigerated for more than one hour*. *Otherwise*, *it would get contaminated with bacteria*. *In our houses*, *we leave the food unrefrigerated for several hours*. *So this tip was very helpful” (FG3P1)*. - *“We felt the urge to save some of the money that we had for the times of need” (FG4P4)*.
	**• Negative** **1. Impact on health** - Emerging physical conditions - Worsening already existing physical conditions	- *“Because of my work in the kitchen*, *I started having a condition in my spine” (FG1P3)*. - *We cannot say the kitchen caused the pains that I have*. *I have been struggling with calcification in my arm joints since 2005*, *so I think the kitchen has definitely worsened this condition*. *And as I said earlier*, *we are getting older*, *and this has also to do with our deteriorating health status” (FG3P1)*.
	**2. Impact on social life** (debatable)	- *“As for me*, *I stopped visiting people*. *If there was a special occasion*, *such for example someone was sick*, *I used to check up on them over a phone call"(FG3P2)*. - *It felt like I was away from people and I personally don’t like to be disconnected from people that I like spending time with*. *I believe in the saying that heaven without people is unbearable” (FG1P3)*.
	**3. Impact on family time** (for women having children)	- *“For me*, *it was a bit difficult in the beginning because I have a young child*. *I used to leave him with my mother*. *Sometimes I felt I was harsh on my child because I was supposed to spend more time with him” (FG3P3)*.
	**Impact of the interruption of CK services** **1. Food insecurity/financial burden** - Financial burden (not attending to family needs such as education and rental fees) - Spending all saved money - Decrease in the amount of food available (dependency on food pots, debatable; some CWs have some sort of family support)	- *“I pay 200USD house rental fees at the end of each month*. *During the two months in which the kitchen was stopped*, *I couldn’t pay the rental charges*. *Now I have to pay 400 USD at once” (FG1P3)*. - *“I spent all the money that I have previously saved” (FG4P3)*. - *“Now we have to reduce our consumption*. *We have no other choice*, *I’m telling you” (FG4P2)*.
	**2. Personal/psychological** - Disruptive to the daily routine - Negative psychological impact	- *“We felt lost in the beginning of the two months in which we stopped working” (FG4P1)*. - *“I felt really hopeless because I had no source of income” (FG4P3)*.
	**3. Type of work:** - Losing convenient work shifts - Losing a nice work environment	- *“First*, *other than the fact that we will have to abandon the altruistic work and leave needy families behind*, *the work shifts were very convenient*. *Would we be able to find such a convenient work shift or type of work*?*” (FG2P1)*. - *“We have not seen each other since one and a half month…we feel lost being far away from each other*, *we feel there’s something missing now” (FG4P1)*.
	**4. Social (Beneficiaries will be suffering)**	- *“When we pass by the beneficiaries on the street*, *they know we work in the kitchen*, *so they start asking when are we going to open the kitchen*? *We tell them hopefully it won’t be delayed anymore and the kitchen’s activity will be resumed again very soon” (FG1P3)*.
	**5. Coping mechanisms** - Need to think of alternatives (searching for a different job) - CWs urged administration not to stop	- *“We will have to search for a new job” (FG3P1)*. - *“We talked to the director of the COOP and we asked her not to stop the kitchen because of the difficult situation of all of us*. *We also talked to the kitchen responsible several times and we discussed with him what actions they can do to sustain the kitchen*. *However*, *he couldn’t do anything about it" (FG1P2)*.
**d. Sustainability of CKs**	**1. Facilitators and opportunities** - Skills - Sense of belonging and ownership - NGO’s can provide funds through new projects - Culturally acceptable type of work Need (CWs need to work, Lebanese families need help) - Employment opportunity for many people Strong will **2. Options:** farmer’s market (*Mouneh*, catering, frozen vegetables, desserts and pastries, frozen *Mezze* items)	- *“We can prepare any type of food*. *For example*, *bread and pastries because we have prepared Manakish many times” (FG1P3)*. - *“As long as the kitchen is still operational*, *we will continue to work even If the municipality established a larger Mouneh house in the village” (FG1P1)*. - *“All NGO’s work for a cause and they receive funds more than municipalities*. *As my colleague mentioned*, *they could gather funds through a fund raising breakfast*, *dinner or festive*. *They get funds through organizing such events” (FG2P1)*. - “*Cooking is a female job*” (FG4P2) - *“They are focusing their efforts on Syrians however there are many Lebanese who are in need more than Syrians*. *I shouldn’t say this because I’m Syrian*, *but Syrians are receiving a lot of assistance*. *If you look around Zahle you would find many Lebanese families whose situation is much worse than that of Syrian refugees who live in ITS” (FG2P1)*. - *“There are many unemployed people who used to ask us whether the kitchen needs more employees” (FG3P3)*. - *“We are very interested in sustaining the kitchen for several reasons which go beyond the mere financial benefit” (FG1P1)*. - *“We might expand our work more*. *We might get funds from our organization and then we can prepare for example desserts and pastries” (FG3P2)*.
	**3. Barriers and threats** - Kitchen administration is the decision maker/lack of support from local authorities/ municipalities - Missing space and tools	- *“The ladies in the kitchen administration are the decision makers” (FG3P1)*. - *“The municipality won’t support us because ‘Caritas’ is willing to produce Mouneh here in the municipality center*. *We wanted to suggest a cooperation with the kitchen administration to sustain our kitchen*, *but they told us to hold on” (FG1P2)*. - *“We need stoves and refrigerators*. *For example we cannot make dairy products such as Labneh and cheese and leave them out of fridge for 2–3 days*. *We need to store them immediately in the refrigerator…” (FG1P3)*.

**Table 2:** Complete list of themes, subthemes and supportive quotes for Syrian refugees' focus groups.

**Main Theme**	**Subthemes**	**Supportive quotes**
**a. Perception towards CKs**	**1. Familiarity** with this concept before coming to Lebanon (during war in Syria)	- *“We got familiar with this concept for the first time during the crisis in Syria*. *We never heard of something like this before” (FG2)*.
	**2. Experience** - Positive and new experience - Would love to transfer this experience to their communities in Syria - Negative perceptions: More transparency, and participation (selection of project beneficiaries by IOCC is unfair, families are not aware of the selection criteria)	- *“The idea of the kitchens is very nice” (FG3)*. - *“If funds were available then yes we would like to transfer this concept to our communities in Syria” (FG1)*. - *“The selection process is unfair*. *There are people who can afford buying food and yet they get registered” (FG3)*.
**b. Impact of CKs**	**Impact of being involved in the CK services** **1. Food and nutrition security** - Fulfilling a dire need for food for all family members especially for children - Opportunity to expand wider variety of food items and consume more quantities - Positive impact on nutritional and health status	- *“We were in dire need for food*, *specially our children” (FG1)*. - *“We used to receive fruits as well as vegetables like tomatoes*, *lemons and cucumbers” (FG2)*. - *“We used to be weak and our children were slim*. *When we started receiving food*, *our health became much better” (FG4)*.
	**2. Financial** - Saving on expenses (food mainly bread, detergents) - Saving money to buy something else (fuel, food, medicine…) - Saving them from falling into debts	- *“The kitchen service decreased our household expenditure” (FG2)*. - *“We were able to save on many products such as bread*, *tomatoes and cucumber to buy something else” (FG3)*. - *“We didn’t borrow food or other non-food products from small shops because we had some money to buy it” (FG2)*.
	**3. Personal/psychological** - Satisfaction, decreased stress associated with having to provide for the family - Saves time and provides opportunity to do other home chores, sleep or go out	- *“Sometimes I have no money to buy food*. *When I was receiving food from the kitchen*, *I didn’t have to worry about this” (FG4)*. - *“We have to do house chores and help our children in their school assignments” (FG3)*.
	**4. Social cohesion** - Unity and agency/equity within the community - Sharing food and equipment, i.e. refrigerators	- *“Sometimes our neighbors used to grill meat and the smell was breath taking*. *So when we all started eating the same food and the same bread*, *I felt satisfied and I enjoyed eating a lot” (FG4)*. - *“Those who have refrigerators share them with their neighbors” (FG1)*.
	**Impact of the interruption of CK services** **1. Financial** - Increase in financial burden (simultaneous with their suspension from the WFP food assistance program, inability to fulfill basic family needs, absence of job opportunities for men during winter time) - Exacerbated debts - Loss of cost opportunity to purchase something else	*- Now we have to purchase all food products that we need” (FG3)*. *- “Most of us opt to borrow food*. *You can ask the shop owner from whom we all borrow bread and vegetables about this” (FG4)*. - *“Before the interruption of service delivery*, *I used to save money to buy something for my children*. *I earn 10*,*000 L*.*L per day*, *which is now barely enough to meet the basic needs of my family” (FG4)*.
	**2. Food and nutrition security** - Drastic decrease in the diversity and quality of food consumed - Negative impact on nutritional and health status of children	- *“We used to consume meat regularly because it was included in almost every dish they delivered to us*. *Now we eat meat less frequently” (FG2)*. - *“We usually take our children to the health centers only when they fall sick*. *Recently they started to look pale and we couldn’t figure out the reason*. *So I took my child to a health center and his blood test showed that he’s anemic” (FG4)*.
	**3. Personal/psychological** - Negative psychological impact (also for children) - Unfair - Having to walk a long distance to buy cheap bread	- *“Indeed it had a negative psychological impact on all of us” (FG1)*. - *“They [the project partners] were unfair with us” (FG2)*. - *“We have to walk a long distance down to El Mina in order to buy 2 packs of bread for 1000 L*.*L instead of buying 1 pack for the same price here” (FG3)*.
	**4. Adopt coping mechanisms** - Saving food for children - Depleting limited assets	- *I only eat food that is enough to keep me alive*. *I save the rest for my children because they need food more than me” (FG3)* - *“I felt distressed because I suddenly stopped receiving the service that I’ve been benefiting from for quite a long time*. *Now I had to economize again” (FG1)*. - *Now If we receive any in kind food assistance we cannot save anything from it for winter time like we used to do before” (FG4)*.
	**5. Social** - Tension at home because of poor household circumstances - Tension with the hosting community - No tension between SR families that are still receiving food and those that were suspended	- *“I have four children*, *together with my husband we are six*. *We have to pay 200$ house rental fees and almost 65$ electricity charges*. *I am left with only 260$ which I have to use wisely and this creates tension inside my home” (FG3)*. - *“Because of the tension between the Lebanese and the Syrians*, *the Syrians were suspended from the WFP assistance program*. *The WFP established something called the “Social Affairs” which is directing the assistance that we are supposed to receive to the Lebanese” (FG3)*. - *“We even share food with each other*. *Families that are still getting food would share it with their neighbors who don’t” (FG3)*.
**c. Sustainability of CKs**	**1. Facilitators & opportunities** - Skills in cooking - Cultural acceptance - Events wedding, funeral but it has to be motivated by external	- *“We once had a wedding ceremony inside the camp and we cooked food in large quantities that day” (FG4)*. - *“If the kitchen was established inside the camp then our families wouldn’t have any problem with us working in the kitchen” (FG1)*. - *“We are interested in working within a CK because we had previous experience where we all cooked together on a wedding ceremony and a funeral inside the camp” (FG4)*.
	**2. Barriers and threats** - Funding (if available, then initiate a CK)	- *“If someone offered to support us in initiating a CK*, *we would all participate*. *However*, *it’s not an easy task for us to suggest a project like this to donors” (FG4)*.

## Discussion

CKs have been previously explored as community-based initiatives aimed at preventing food insecurity, promoting nutrition education, reducing social isolation, and improving the cooking and food management skills of low-income families [5]. The present study is the first, to our knowledge, to examine the impact of CKs as a form of food assistance among low-income families within a context of conflict and displacement.

Findings from this study show that CKs can alleviate food insecurity and enhance dietary diversity, while fostering social cohesion among refugees and host communities within a protracted crisis setting. It is important to interpret these results in light of the growing literature that emphasizes the challenges with interventions being mobilized to address food insecurity in protracted crises settings. According to Maxwell et al (2012), these challenges include: 1) extreme levels of food insecurity witnessed within conflict-affected settings, 2) the short-term nature of interventions commonly mobilized in such contexts that do not necessarily induce sustainable long-term improvements, 3) weak investments and limited international funding as conflicts last for extended periods of time, and 4) the incapability or unwillingness of the local governments and states to contribute to alleviating food insecurity [21].

In Lebanon, the protracted crisis had dire consequences on the food security status and livelihood of SRs and the Lebanese host community equally. Deteriorating economic, social and health situations are worrying consequences. About 75% of SRs and 27–30% of Lebanese (1.5 million) are considered vulnerable; some are living below the poverty line of $3 per day [22]. Further, the protracted crisis is creating worrying signs of tensions between SRs and the host community. There is also growing evidence highlighting that food insecurity represents a challenge to the Lebanese communities in both urban [17] and rural settings [23]. In fact, several reports have been published to date highlighting the increased tension between the SR and Lebanese host communities due to competition over limited employment opportunities and access to basic services, including food, water, shelter, education, and health services [23–26]. The present study provides an example of a successful and unique initiative that had a positive multi-level impact on the food security and overall wellbeing of individuals from both the Syrian refugee and host communities.

Overall, women participants from both groups (CWs and SR community) expressed positive perceptions and experiences towards the CKs and its impact on their household finances and food security status. Participants reported that kitchens allowed them to stretch their budgets, protect them from accruing debt, and increase their food resources through receiving food aid in the form of food pots. In addition, CWs reported learning new skills related to preparation of new foods and in large quantities, ensuring safe food handling, and better financial management. Both groups also reported that receiving food from the CKs allowed them to have a more diverse diet that includes more animal protein sources, which contributed to better health and nutritional status among their family members, including children. Previous studies have shown that participation in CKs can increase the access to nutritious and more diverse food [1,14,27] and improve the capacity of participants to address some of their food security concerns through learning budgeting and cooking skills and how to save money [28–30], however, evidence is still inconclusive as to whether participation in CKs can have a substantial and consistent impact on income-related food insecurity [1,5]. Researchers have argued that CKs can have limited potential to resolve food security challenges rooted in chronic and severe poverty given that kitchen services cannot induce any fundamental changes in household economic conditions [1,14,29,31]. In the case of the present study, CWs reported receiving cooked food in addition to monthly salaries in return for their work in the CKs to prepare meals in bulk amounts, which may have contributed to their improved perception of food security and financial stability. This stands in contrary to CK participants in other studies who only benefited from the cooked food they took home, but did not receive a steady income or salary [1,2].

The inconsistent impact of CKs on the food security status of participating housheolds across studies can also be attributed, in part, to the diversity of CK modalities adopted in various settings. CKs may differ in their purposes, management, funding, and services offered to participants. For example, some CKs focus on nutrition education, cooking and food preparation skills, while others emphasize the social aspects of the CKs, as self-help grass-roots programs, that aim at empowering women to become advocates of change within their communities [2,3]. The involvement of social workers and public health professionals has also been noted in some CKs, while community volunteers assume leadership positons in other kitchens [2]. In addition, funding for CKs vary considerably from one setting to another as some kitchens are state-funded or supported by international food aid, non-governmental organizations, and project grants, while others are based on self-help, grassroots actions and depend solely on contributions of participants [1–3].

Women from both groups in the present study highlighted the psychological and social health benefits of the CKs. These benefits included higher satisfaction and peace of mind for CWs and SRs, who had to worry less on how to secure food for their families, which in turn allowed them to focus on other activities, home chores, and socialization. The CK was a source of ‘strength and motivation’ for CWs as they felt more financially independent, and the kitchens served as venues for socialization with other participants. In addition, CWs perceived the work environment as positive and overall joyful allowing for the provision of psychological and emotional support between participants. Among the SR community, participants felt a sense of agency and unity as they all received the same food pots from the CKs. This research confirms results from other studies conducted in other settings showing that social support was an almost universal element of the CKs [32]. Those kitchens were previously commended for promoting social and emotional support through increased social interaction among participants, creating a healthy environment within the kitchens, and reducing feelings of isolation [5,27,28,32]. According to Tarasuk and Reynolds (1999), participation in CKs is fundamentally a social experience and is even considered as a “social outing” for adults from low-income families [1], who may otherwise have limited opportunities to socialize due to their scarce resources and childcare responsibilities. In addition, the cooperative nature of preparing food within these kitchens as a group creates a social atmosphere that enables the sharing of ideas and information among participants [1,32].

An interesting and unique attribute of the CKs explored in the present study was the role it played in facilitating social cohesion between the refugee and Lebanese host communities. The CK served as a friendly environment that strengthened the social cohesion and rapport between CWs, who were either SRs or from the Lebanese host communities. In addition, CWs reported feelings of empathy towards the SRs given all the hardships that they were enduring and they felt satisfied with the altruistic nature of the CK work in providing food and saving lives. CKs have been previously noted to reach and engage diverse population groups that face great health inequities, including low income families, those with disability, indigenous populations, and immigrants [4,29,33]. However, the present study provides unique insight as to how CKs can go beyond being mere venues for socialization and sharing of knowledge and skills to become sites that enable social transformation through reducing social tensions and increasing feelings of unity and equity. This is particularly important when dealing with sub-population groups in harsh conflict and displacement settings that may otherwise be competing for limited resources and livelihood opportunities.

It is worth noting that the overall positive impacts of the CKs were almost all reversed once the CKs’ operation and services were interrupted during the summer of 2017. CWs and SRs reported the negative repercussions that this interruption had on their household finances, food security status, and their overall psychological health. Unfortunately, it was noted from the discussions with the SR participants that social tension re-emerged between the refugees and host community upon the interruption of the CK services. This tension was explained in part by the confusion that SR participants had as to what caused the interruption of the CK services and their misperceptions that the humanitarian assistance provided to SRs was re-channeled to the Lebanese host community.

One of the main themes that emerged from our discussions with CWs was the motivation to work in the CKs. The type of work, which was convenient and socially and culturally-acceptable for women, was one of the motivating factors. Another main driver for participating in the CKs was financial allowing CWs to provide food and basic needs for their families without having to rely on charity. Many CWs also reported that their motivation to participate in the CK goes beyond their financial need to work, as they considered this type of work to be altruistic in nature and was an opportunity for self-development and feeling of self-worth. In fact, CWs were aware that the main purpose of the CK was to provide food for SRs living in ITSs within nearby areas, which gave CWs a sense of satisfaction that they can contribute to improving the lives of others even with their limited means. Similar to our study findings, Engler-Stringer [14] and Tarasuk & Reynolds [1] noted that CK participation increased feeling of dignity and reduced sense of shame or stigma, among low-income families in Canada, as compared to food banks or other charitable food assistance programs [1]. Studies from Peru and other Latin American countries also support the role that CKs had in empowering women as they were able to challenge community power structures and organize community development activities that extended meal services to most needy families within their communities [3].

Another important theme that emerged from the discussions is the strong interest of CWs and SRs in the sustainability of the CKs. Both groups identified a number of facilitators and opportunities that can assist in the continuity of the kitchens, such as learned cooking skills and strong sense of belonging to the CK among CWs. In addition, the SR group showed interest in initiating similar kitchens within their ITSs that can help serve a large number of families within their community and can also be contracted to prepare food for special occasions, such as weddings and funerals. Nevertheless, the lack of personal funds to support such kitchens was one of the main barriers identified by women from both CWs and SR groups, in case funding for CKs from donor agencies and NGOs got interrupted. In addition, both CWs and SRs lacked training on entrepreneurial and management skills and felt that they were completely dependent on the availability of funding from the donor agencies for the survival of their CKs.

One would argue that CKs need be designed and operated in a more participatory approach that ensures their sustainability and the development of the local communities where kitchens operate beyond the short-term nature of emergency programs and humanitarian interventions. In addition, the design of such social welfare and public health interventions should resist developmental imposition from above and avoid creating dependency on external funding from international or local donors [34].

### Strengths and limitations

The present study has a number of strengths. To our knowledge, this study is the first to qualitatively explore the impact of CK within a conflict-affected setting. The purposeful recruitment approach adopted in the present study allowed our research team to assess the impact of CK operating in different geographical regions in Lebanon on low-income participants. In addition, data triangulation was possible through exploring the CKs from the perspective of the CWs and the SR beneficiaries. We also adopted a number of measures to increase the trustworthiness of the study and its results including increasing credibility, reflexivity, and objectivity. Nevertheless, the study findings need to be interpreted in light of few limitations. Interviews were not conducted with key informants, such as representatives from the administration of CKs and humanitarian agency involved in the planning, implementation, and funding of the program. The latter could have allowed for a more comprehensive examination of the CK impact. Given the sensitive nature of the topic and study population, the risk of social desirability bias cannot be ruled out; CK workers and SR beneficiaries may have overstated the positive impact and benefits of the CKs. Nevertheless, every attempt was exerted by the research team to ensure impartiality and reflexivity at each stage of the study.

## Conclusion

Findings from the present study show that CKs can be promising social welfare and public health strategies to alleviate food insecurity and foster social cohesion among refugees and impoverished host communities in Lebanon. Future studies need to further examine the long-term impact, sustainability, and cost-effectiveness of CKs as food assistance modalities within conflict and displacement settings. In addition, the role that these kitchens can play in women empowerment and local community development beyond immediate food assistance is an area that warrants further attention.

## Supporting information

S1 Table Topic guide for focus group discussions with community workers. (DOCX)

S2 Table Topic guide for focus group discussions with syrian refugees. (DOCX)

S3 Table COnsolidated criteria for REporting Qualitative research (COREQ) checklist. (DOCX)
