# Supplementary material for: Perceived impact of community kitchens on the food security of Syrian refugees and kitchen workers in Lebanon: Qualitative evidence in a displacement context
Source: PLoS One. 2019 Jan 25;14(1):e0210814. doi: 10.1371/journal.pone.0210814 (PMC6347439; doi:10.1371/journal.pone.0210814)
Supplement: S1 Table — (DOCX) [file pone.0210814.s001.docx]

**S1 Table Topic guide for Focus Group Discussions with Community Workers**

| **Concept** | **Main question** | **Probing questions** |
| --- | --- | --- |
| **Introduction** | **I am interested in understanding what motivated you to become a community worker in CK services?** | **What characteristics of CK services influenced your decision?**  **What is the personal added value of working in CK services?**  **Describe if there is any negative influence of working in a CK on your personal life.**  **What is the family and social added value of working in CK services?**  **Describe if there is any negative influence of working in CK on your family** |
| **Service preparation and delivery** | **Describe the process of service preparation and delivery?** | **Walk me through the process of what happens from the moment you start planning and preparing the food until the service is delivered to households**  **What challenges you encounter during that process?**    **What factors facilitate this process?**  **In your opinion, what measures are needed to improve the service**  **preparation and delivery?** |
| **Spatial-Temporal** | 1. **What do you think of the location of the CK?** 2. **What about the kitchen facility?** 3. **Tell me about transportation to reach the CK** 4. **What about the time commitment?** | **Is it easy to access?**  **Do you have to travel a long distance to reach the CK?**  **Describe the kitchen facility.**  **What elements facilitate your work?**  **What about the challenges?**  **What do you use as transportation to get to the CK?**  **What are the challenges?**  **What are the opportunities?**  **How long is your engagement in the CK?**  **How this commitment is affecting your personal life?**  **How this commitment is influencing your family life?** |
| **Economic** | **Now, I need to ask about the financial impact of CK services. Tell me about how being a CW in a CK influenced your financial status.** | **How is the money you receive helping you personally?**  **How is it helping your family?**  **Is it enough to cover your expenses?**  **Does receiving food from the CK allow you to save on your home finances?** |
| **Social** | **Tell me about the relationships you developed inside the CK** | **Describe the nature of the relationship you have with your colleagues at work**  **Describe the nature of the relationship with your colleagues outside work, socializing, supporting etc…**  **In your opinion, what is the social added value of working in a CK?**  **What about your relationship with the CK administration?**  **What facilitates this relationship?**  **What challenges this relationship?** |
| **Personal** | 1. **Tell me about the impact of working in the CK on your personal health status?**      1. **Tell me about the learning process in the CK** 2. **How does working as a CW affect you psychologically?** 3. **What happens if the delivery of CK service stops?** | **Describe how do you feel after a day of work at CK facility?**  **Did you experience an onset of any disease or medical condition as a result of your work in the CK?**  **Did you receive any training to be part of the CK? Please explain.**  **What did you learn as a result of your involvement in the CK?**  **How does it contribute to your nutritional knowledge?**  **What new skills did you acquire?**  **Tell me how do you feel towards yourself?**    **How is it going to affect your life style?**  **Your home expenses?**  **Your psychological status?** |
