# Supplementary material for: Perceived impact of community kitchens on the food security of Syrian refugees and kitchen workers in Lebanon: Qualitative evidence in a displacement context
Source: PLoS One. 2019 Jan 25;14(1):e0210814. doi: 10.1371/journal.pone.0210814 (PMC6347439; doi:10.1371/journal.pone.0210814)
Supplement: S2 Table — (DOCX) [file pone.0210814.s002.docx]

**S2 Table Topic Guide for Syrian Refugees Focus Group**

| **Concept** | **Main question** | **Probing questions** |
| --- | --- | --- |
| **Introduction** | **I am interested in understanding how do you perceive being a recipient of CK services?** | **How CK services did influence you personally?**  **How CK services did influence your family?** |
| **Service delivery** | 1. **Describe the process of service delivery?** 2. **Describe the type of food you receive?** 3. **Describe your relationship with individuals delivering the service** | **Walk me through the process of what happens from the moment CK comes to deliver the food.**  **What challenges you encounter during that process?**    **What factors facilitate this process?**  **How the service delivery can be improved?**  **Is it enough?**  **What about the variety?**  **The nutritional value?**  **What do you think of the food presentation?**  **Do you have any family member who has a chronic disease such as diabetes? Is the food appropriate for this family member?**    **Do you like the food?**  **Is this the type of food you used to consume in Syria?**  **How can the food provided be improved?**  **What do you think of them?**  **Tell us how do they interact with you?** |
| **Spatial-Temporal** | 1. **When do you usually receive the food?** 2. **What do you think of the location of the service delivery?** | **Is this is a good timing?**  **When do you usually consume the food?**  **Where do you usually store the food?**  **Do you have to walk a long distance to receive the food?**  **Is this location appropriate for all weather conditions?** |
| **Economic** | **Tell me how CK services affect your financial status?** | **Do you think the food you receive is sufficient for your family needs?**  **What happens when the food you receive is not enough?**  **How do you feel towards buying supplemental food?**  **Do you think that the money you save from receiving CK services enable you to buy something else? Please elaborate** |
| **Social** | **Would you consider working in a CK** | **Why or why not?** |
| **Personal** | **What happens if you stop receiving CK services?** | **How is it going to affect your food intake?**  **Your home expenses?**  **Your social and psychological status?**  **Your family situation?** |
|  | **Any other thoughts that you would like to share with us today?** |  |
